# Does Knowing More Relate to More Anxiety? Psychological Factors Associated With Anxiety About Radiofrequency Electromagnetic Fields and the Role of Information Provision in Risk Communication

**DOI:** 10.1002/bem.70070

**Published:** 2026-10-19

**Authors:** Ruohan Wen, Sachiko Yamaguchi‐Sekino, Masao Taki, Teruo Onishi, Michio Murakami

**Affiliations:** ^1^ Electromagnetic Compatibility Laboratory, Radio Research Institute, National Institute of Information and Communications Technology (NICT) Koganei Tokyo Japan; ^2^ Radio Research Institute, National Institute of Information and Communications Technology (NICT) Koganei Tokyo Japan; ^3^ Center for Infectious Disease Education and Research (CiDER) The University of Osaka Suita Osaka Japan; ^4^ EIPM Center The University of Osaka Suita Osaka Japan

**Keywords:** anxiety, intervention, multilevel analysis, radiofrequency electromagnetic fields, risk communication

## Abstract

As radiofrequency electromagnetic fields (RF‐EMFs) become increasingly embedded in everyday life, understanding public responses to RF‐EMFs and developing effective risk communication strategies are essential. In this study, we examined the psychological factors associated with anxiety concerning the use of technologies that rely on RF‐EMFs and the effects of information provision as a form of risk communication. In the baseline analysis, multilevel modeling of anxiety across six common RF‐EMF sources revealed that both subjective exposure perceptions and confidence in those perceptions were independently associated with anxiety. While heightened awareness of RF‐EMFs was associated with higher anxiety, more objective knowledge of RF‐EMFs was associated with lower anxiety. In the intervention analysis, although information provision increased anxiety overall, it reduced anxiety among those with initially high levels of concern. It also affected a range of related cognitive factors, including subjective exposure perceptions, confidence in those perceptions, awareness, and knowledge. These findings demonstrate the complex psychological impact of information provision on anxiety about RF‐EMFs and related cognitive factors and provide practical insights for designing more effective risk communication strategies.

## Summary

- This study investigated anxiety about RF‐EMFs among the general public and examined its malleability through information provision by using large‐scale panel survey data from Japan.
- Both subjective exposure perceptions and confidence in those perceptions were independently associated with anxiety.
- Information provided through risk communication not only affected anxiety but also a range of related cognitive factors.

## 1 Introduction

Anxiety is typically characterized by a sense of tension and worrying thoughts about potential future threats (Grupe and Nitschke 2013). It is often regarded as an anticipatory emotional response that is elicited by uncertainty and perceived risk (Loewenstein et al. 2001). In modern society, radiofrequency electromagnetic fields (RF‐EMFs) have become indispensable to everyday infrastructure. For example, signal transmission and reception in smartphones and wireless routers all rely on RF‐EMFs. However, RF‐EMFs cannot be seen, heard, or smelled, making them imperceptible to human senses. This imperceptible feature of RF‐EMFs may arouse some individuals' anxiety about their effect on health (cf. Skotnes et al. 2021; Slovic et al. 2016).

Regarding the effects of RF‐EMFs on the human body, the International Commission on Non‐Ionizing Radiation Protection (ICNIRP) has reported that, to date, their biological effects have only been identified as short‐term exposure, which are known as stimulation effects (< 10 MHz) and thermal effects (> 100 kHz) (International Commission on Non‐Ionizing Radiation Protection 2020). On the basis of this scientific evidence, the ICNIRP has issued safety guidelines regulating exposure levels (International Commission on Non‐Ionizing Radiation Protection 2020). As for long‐term effects, several groups have focused on the potential carcinogenicity of RF‐EMFs, particularly their association with brain tumors, but no reproducible adverse outcomes have been identified (Karipidis et al. 2024). Nevertheless, laypeople are commonly assumed to evaluate risks by using heuristics and other informal thought processes rather than scientific evidence (Paek and Hove 2017). Misconceptions about RF‐EMF exposure and health effects are common among the public and are associated with higher perceived risk and mitigation behaviors (Wiedemann et al. 2017). Some individuals may also develop heightened fear regarding the use of RF‐EMF‐based technologies (e.g., Frith et al. 2023; Soler 2022).

Although previous research suggests that risk perception about RF‐EMFs among the general public is typically moderate and often of low daily salience compared with other environmental health hazards (Timmons et al. 2024), the widespread use of RF‐EMFs and their foundational role in modern communication infrastructures mean that such concerns can still become social issues in specific contexts. For example, in Switzerland, thousands of citizens launched a referendum campaign opposing the rollout of 5 G technology (Keystone 2019). In Japan as well, several incidents have arisen in which plans to deploy 5 G base stations have sparked strong public concerns about the health risks posed by radio waves, ultimately forcing the authorities to abandon the projects. These issues highlight the importance of risk communication regarding RF‐EMFs.

To inform more effective risk communication, previous research has primarily examined risk perception of RF‐EMFs among the general public, and its associated psychological factors. Among these, individuals' subjective perceptions of exposure have consistently been identified as a critical factor correlated with RF‐EMF risk perception (e.g., Freudenstein et al. 2015a, 2015b; Koh et al. 2020; Ramirez‐Vazquez et al. 2019; Seo et al. 2020). Specifically, when individuals perceive themselves as being more strongly exposed to RF‐EMFs, regardless of whether this perception is realistic or based on objective evidence, they are more likely to perceive greater risk.

However, existing studies have highlighted the public's limited understanding and knowledge regarding RF‐EMF exposure. For example, Freudenstein et al. (2015a) pointed out that people generally lack knowledge of RF‐EMF strength and tend to rely on heuristic judgments when forming perceptions. Link et al. (2025) similarly suggested that individuals' perceptions of RF‐EMF exposure from mobile phone use are more often affected by emotion than by objective facts or evidence. Together, these findings suggest that public judgments about RF‐EMFs are often formed under substantial uncertainty rather than on the basis of well‐informed risk assessments. Under such conditions, public judgments about risk are likely to contain a substantial affective component, such as anxiety (Loewenstein et al. 2001). This makes anxiety a particularly relevant outcome to examine, although previous research has rarely approached it. Moreover, another implication of this uncertainty in exposure perception is that individuals may also differ in how confident they are in that judgment. Nevertheless, previous studies have rarely examined confidence in one's subjective exposure judgment as a distinct factor associated with public responses to RF‐EMFs.

At the same time, the public's limited knowledge of RF‐EMFs highlights the potential malleability of risk perception about RF‐EMFs. As empirical evidence, Ramirez‐Vazquez et al. (2019) provided participants with wearable devices to measure their actual RF‐EMF exposure and offered feedback. Their findings showed that receiving such feedback significantly reduced perceived risk compared with those who did not receive exposure data. Similarly, Zeleke et al. (2021) found that participants who received information about their actual RF‐EMF exposure reported greater confidence in protecting themselves.

However, the role of information provision is unlikely to be limited to such immediate endpoints. Because RF‐EMF risk communication typically provides information about exposure levels, health evidence, and the technological role of RF‐EMFs, it may also reshape broader cognitions, such as awareness of the electromagnetic environment, understanding of biological effects, and perceived necessity of these technologies in daily life. These post‐intervention cognitions may be further associated with subsequent affective responses. However, such inclusive effects of information provision have rarely been considered in previous research, limiting our ability to comprehensively evaluate its effectiveness and to guide future strategies.

Against this background, the present study has two aims. First, drawing on the perspective of “risk as feelings” (Loewenstein et al. 2001), we focus on anxiety about RF‐EMFs among the general public and examine the psychological factors associated with it. Second, we examine whether anxiety and these associated factors are affected by information provision in risk communication. Specifically, we focus on anxiety experienced when using common devices that rely on RF‐EMFs in daily life. In the present study, anxiety is conceptualized broadly to include related affective states such as worry, concern, and a sense of tension or discomfort associated with potentially negative outcomes. To address these aims, we first develop a comprehensive model regarding individuals' anxiety about RF‐EMFs, incorporating a range of cognitive and individual factors from multiple perspectives. On the basis of this model, we then examine how information provision affects both anxiety and its related factors. The following two sections present the theoretical model and the content and structure of the information provision used in this study.

### 1.1 Psychological Factors Associated With Anxiety About RF‐EMFs

We construct the theoretical model of the psychological factors associated with anxiety about RF‐EMFs, with particular attention to their relevance to anxiety and whether they may be influenced by information provision. These factors are organized into three categories: (1) core RF‐EMF cognitive factors, which refer to individuals' attitudes toward specific RF‐EMF environments encountered in daily life; (2) knowledge‐based factors, which refer to individuals' general knowledge and understanding of RF‐EMFs, as well as the ways in which such understanding is formed; and (3) other individual‐specific factors, which capture individual‐level differences that are not assumed to be directly related to anxiety but may serve as relevant covariates with potential background influences. Among these categories, the core RF‐EMF cognitive factors and some of the knowledge‐based factors are assumed to be potentially influenced by information provision.

Given the widespread use of RF‐EMFs in modern life, individuals are exposed to various RF‐EMF sources, such as mobile phones, Wi‐Fi routers, and Bluetooth devices. While people may hold different risk perceptions depending on the source, their personality traits may lead to similar cognitive tendencies across different RF‐EMF sources, and attitudes may also be generalized across different RF‐EMF sources (Freudenstein et al. 2023). To capture these within‐individual variations in attitudes toward different RF‐EMFs, we applied repeated measurements and adopted a multilevel modeling approach for core RF‐EMF cognitive factors. In contrast, knowledge‐based and individual‐specific factors were measured only at the person level and did not vary across RF‐EMF types. The following subsections provide detailed descriptions of the variables examined in this study.

#### 1.1.1 Core RF‐EMF Cognitive Factors

We begin by focusing on core cognitive factors related to RF‐EMFs. Previous studies have emphasized the impact of subjectively perceived exposure to RF‐EMFs as a strong indicator of risk perception (e.g., Freudenstein et al. 2015a, 2015b; Koh et al. 2020; Ramirez‐Vazquez et al. 2019; Seo et al. 2020). Therefore, it could also be expected that a higher subjective exposure perception indicates stronger anxiety. In the present study, we define individuals' perceived level of exposure to various RF‐EMF sources commonly encountered in daily life as *subjective exposure perception*, which is distinguished from actual, objectively measured exposure.

However, because the general public often lacks reliable means to accurately assess RF‐EMF intensity or exposure levels, such perceptions may not rest on a solid evidential foundation (e.g., Freudenstein et al. 2015a). In other words, individuals may differ in the degree of confidence in their exposure perceptions. Previous research has suggested that greater certainty in risk may be associated with stronger anxiety (Gu et al. 2020). Accordingly, we define the extent to which individuals feel confident about their exposure as *exposure perception confidence* and examine its relationship with anxiety about RF‐EMFs.

In addition, we consider individuals' perceptions regarding the necessity of RF‐EMFs. Previous studies in risk perception have shown that recognizing the necessity of the use of technology can mitigate perceived risks (Slovic 1987). Accordingly, we include *perceived necessity* toward RF‐EMFs as an additional cognitive factor in the model.

#### 1.1.2 Knowledge‐Based Factors

Next, individuals' general understanding of RF‐EMFs is likely to affect their attitudes toward the use of these technologies. To capture this dimension, we considered three aspects: general awareness, scientific knowledge, and the ways in which individuals acquire information.

Although RF‐EMFs are imperceptible to the human senses, they are pervasive in modern environments. To capture this dimension, we first examine individuals' awareness of RF‐EMFs—that is, their general awareness of the existence of RF‐EMFs in daily life—and assess whether greater awareness is associated with heightened anxiety.

Second, scientific knowledge of RF‐EMFs is another potentially important factor (Freudenstein et al. 2015a). Public anxiety and perceived risks are often driven by misunderstandings or misinformation about the biological effects of RF‐EMFs (e.g., Rowley and Mazar 2021; Vaupotič et al. 2025). Therefore, a higher level of objective knowledge about RF‐EMFs may help evaluate potential risks more realistically and reduce anxiety.

Third, we also explore two additional factors: information exposure and information seeking. These reflect the degree to which individuals passively encounter or actively search for information related to RF‐EMFs. In the age of social media, people are increasingly exposed to a wide range of information, which can subtly affect their attitudes toward health and technology (e.g., Dashtipour et al. 2021). Thus, both information exposure and seeking may indicate individuals' risk perceptions and emotional responses regarding RF‐EMFs.

#### 1.1.3 Other Individual‐Specific Factors

Finally, we consider individual‐specific characteristics that may be associated with anxiety about RF‐EMFs. Previous research has suggested that individuals may show similar tendencies in their perceptions of different hazards (Hohl and Gaskell 2008). Accordingly, we included participants' perceived carcinogenic risks of three dietary behaviors as covariates: eating red meat, eating pickled vegetables, and drinking coffee. These dietary behaviors are common in daily life in Japan and are frequently debated in public owing to potential cancer risks. According to the monographs issued by the International Agency for Research on Cancer (International Agency for Research on Cancer 2025), red meat is classified as Group 2 A (probably carcinogenic to humans), pickled vegetables as Group 2B (possibly carcinogenic to humans), and coffee drinking as Group 3 (not classifiable as to its carcinogenicity to humans). We examined whether higher perceived risks of these dietary behaviors were associated with greater anxiety about RF‐EMFs.

We also account for demographic characteristics, which previous research has consistently shown to be associated with anxiety (e.g., Freudenstein et al. 2015c; Koh et al. 2020; Zeleke et al. 2021). For example, females and older adults tend to report higher levels of concern. Accordingly, the present study includes age, gender, and education level as control variables.

All the factors discussed above are considered potential indicators of anxiety about RF‐EMFs. The theoretical model proposed in this study is illustrated in Figure 1.

**Figure 1:**
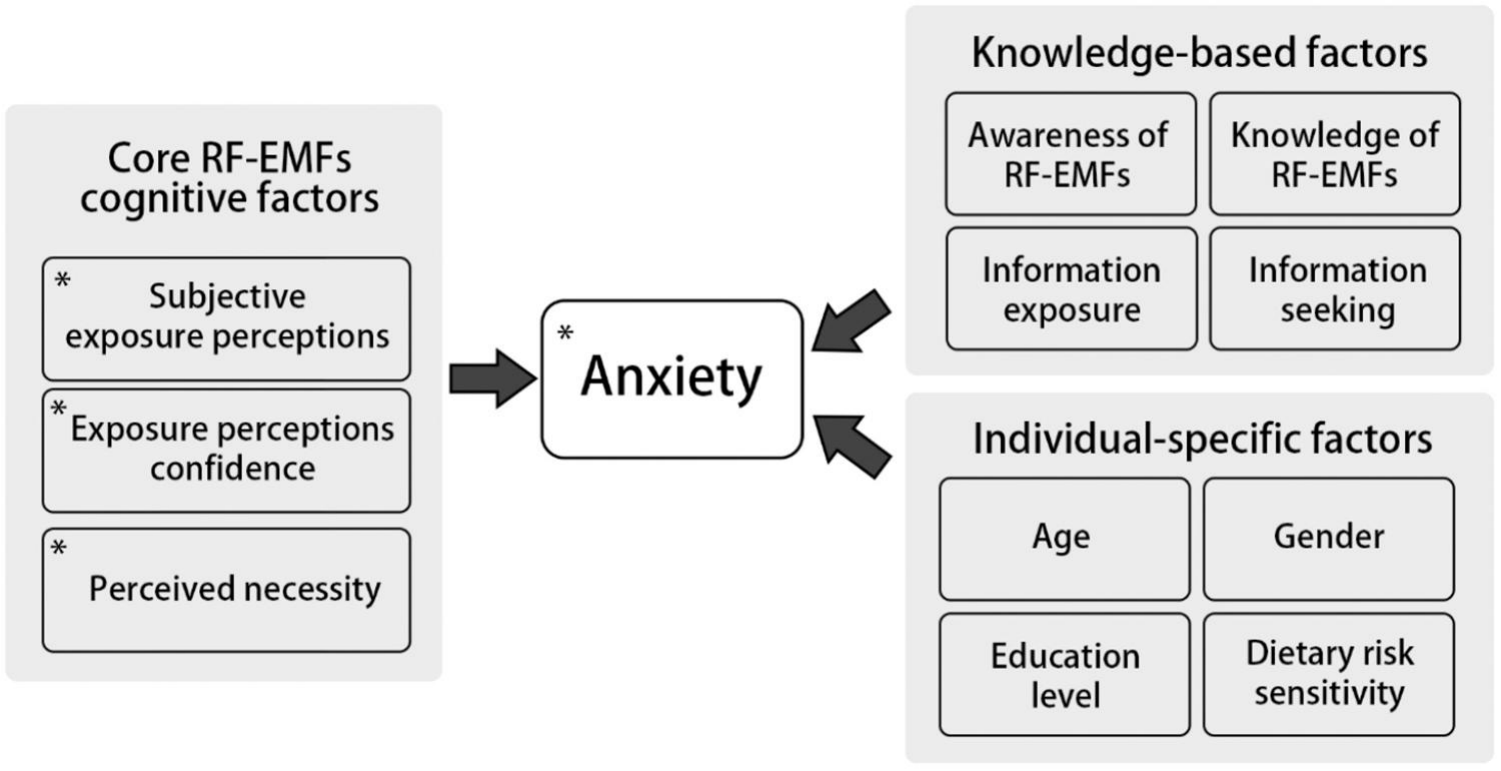
Theoretical model in baseline analysis. * multi‐level data.

### 1.2 Examination of Information Provision

Next, we investigated whether information provision had an effect on anxiety about RF‐EMFs and related psychological factors. In previous studies, various approaches to RF‐EMF information provision were proposed and tested. For example, some provided participants with wearable devices that measured actual RF‐EMF exposure and offered personalized feedback (e.g., Ramirez‐Vazquez et al. 2019). Others provided informational materials developed by expert committees, presenting scientific evidence and regulatory viewpoints (e.g., Cousin and Siegrist 2011; Frey 2021; Zeleke et al. 2021). Based on the types of information provided to participants in these previous studies, we developed multifaceted information that includes basic facts about RF‐EMFs, such as exposure levels in daily life and common biological effects of exposure. We aim to evaluate whether providing such information can not only reduce anxiety about RF‐EMFs but also alter the related psychological factors of this anxiety, thereby contributing to the literature on effective risk communication strategies.

Extending the psychological framework developed through baseline analysis, we propose an intervention model, as illustrated in Figure 2. This model focuses on five factors associated with anxiety about RF‐EMFs: subjective exposure perception, confidence in exposure perception, perceived necessity, awareness of RF‐EMFs, and knowledge of RF‐EMFs. The model consists of two steps. In Step 1, we examine how each of these five factors at Time 1 (T1), together with the intervention, affects each corresponding factor at Time 2 (T2), separately. In Step 2, we then examine how anxiety at T1 and the intervention affect anxiety at T2, after controlling for these five T2 factors.

**Figure 2:**
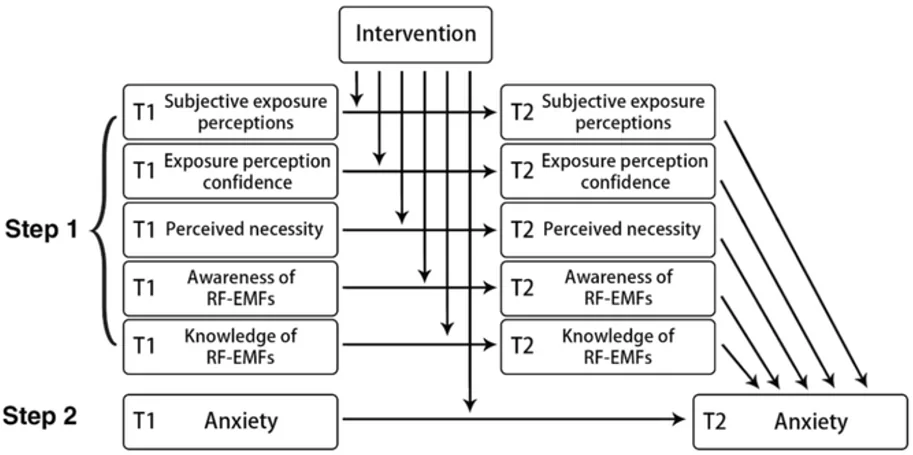
Theoretical model in intervention analysis.

### 1.3 Current Study

To examine the two models, we conducted a baseline analysis and an intervention analysis based on a large‐scale panel survey. Two previous publications have utilized the same dataset (Yamaguchi‐Sekino et al. 2024; Yamaguchi‐Sekino et al. 2025). Yamaguchi‐Sekino et al. (2024) primarily conducted a cluster classification of individuals based on their risk perception feature and reported the differential effects of information provision across these groups. Yamaguchi‐Sekino et al. (2025) focused on identifying individual‐level factors that influence the effectiveness of information provision.

In contrast, in this study, we take a re‐analytical approach to provide a more in‐depth understanding of anxiety about RF‐EMF. Specifically, we first explore the psychological factors associated with anxiety at the baseline stage, and then assess how anxiety and these factors change following information provision.

## 2 Methods

### 2.1 Study Design

In this study, we conducted a secondary analysis using anonymized open data obtained from our previous research (Yamaguchi‐Sekino et al. 2024). This research was approved by the Research Ethics Review Committee of the National Institute of Information and Communications Technology (No. N230042301). The overall research design is illustrated in Figure 3. First, a large‐scale baseline survey was conducted across Japan in October 2023 to assess individuals' perceptions of various RF‐EMF‐related activities, forming the T1 data. Five weeks later, a follow‐up survey was conducted to evaluate the effectiveness of the information provision, forming the T2 data. In the intervention survey, participants were randomly assigned to either an intervention group or a control group, without being informed of their assignment. Prior to the survey, participants in the intervention group were given access to a specially developed website containing information about RF‐EMFs, while the control group received no such information.

**Figure 3:**
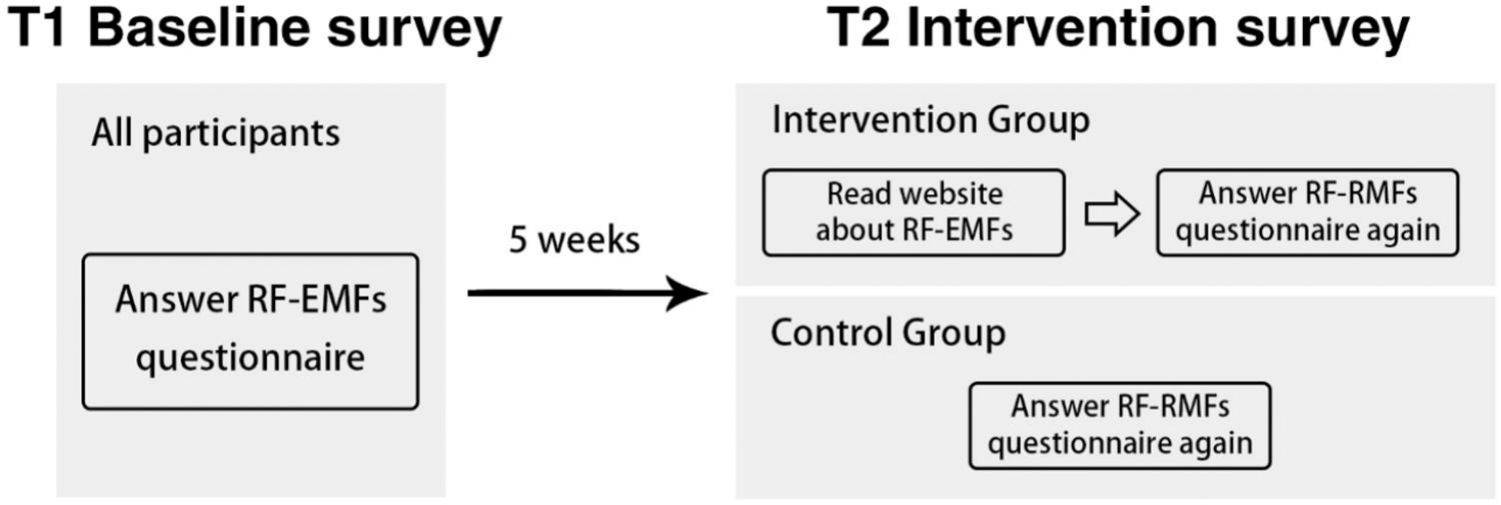
Study design.

Participants were recruited under randomized and blinded conditions through a commercial research panel. The questionnaires were written in Japanese, and the target population consisted of Japanese individuals or residents with sufficient proficiency in the language. Quota sampling for the general Japanese population was employed to ensure that the age and gender distribution of the sample matched that of the national census. Once the required number of respondents for each age and gender group was reached, recruitment was automatically closed. The participants provided their written informed consent to participate. Details of the questionnaires used in both surveys are described in the following sections.

### 2.2 Baseline Survey

The first survey was aimed at assessing the public's baseline attitudes toward RF‐EMFs. The questionnaire included the following parts. The English translation of the items is available in the Appendix materials.

#### 2.2.1 Core RF‐EMF Cognitive Factors

Participants were asked about their attitudes toward the use of six common RF‐EMF‐based technologies selected to represent a broad range of everyday RF‐EMF‐related sources: watching television or listening to the radio, using non‐5G mobile phones, using 5 G mobile phones, using Wi‐Fi, using microwave ovens, and using Bluetooth wireless earphones. For each technology, participants evaluated: (1) their subjective perception of RF‐EMF exposure from the device in their living environment (1 = completely absent, 5 = very strong); (2) their confidence in this perception (1 = not confident at all, 4 = very confident); (3) the perceived necessity of using the device in daily life (1 = not necessary at all, 4 = very necessary); and (4) the anxiety they experienced when using the device (1 = not anxious at all, 4 = very anxious).

#### 2.2.2 Knowledge‐Based Factors

We first assessed participants' awareness of radiofrequency‐related sources such as mobile phones, Wi‐Fi, television, radio, and Bluetooth devices in their daily environment, rated on a 4‐point scale ranging from 1 (not aware at all) to 4 (strongly aware).

Next, participants were asked whether they had been exposed to information about RF‐EMFs (e.g., their properties, intensity, or safety). Responses were recorded using four categories: never, passive only (e.g., happened to see it), active only (e.g., searched for it on the internet), or both. These responses were recoded into two binary variables: information exposure (1 = passive only or both) and information seeking (1 = active only or both).

To objectively assess participants' knowledge of RF‐EMFs, we developed 10 statements regarding their properties and characteristics (e.g., “The strength of radio waves generally decreases as you move away from the source”; “Natural phenomena do not generate radio waves”). Participants were asked to indicate whether each statement was true or false, with an additional “don't know” option. These items were reviewed by experts with extensive experience in RF‐EMF communication and education to ensure their validity. The number of correct responses (excluding incorrect and “don't know” answers) was used as an index of participants' objective knowledge of RF‐EMFs.

#### 2.2.3 Individual‐Specific Factors

Regarding perceived carcinogenic risk in daily dietary behaviors, participants rated the following three behaviors: eating red meat, eating pickled foods, and drinking coffee. Each was rated from 1 (no risk at all) to 6 (very high risk). The average of the three ratings was used to represent participants' dietary risk sensitivity.

Finally, demographic information was collected, including gender, age group, and education level. Age groups were categorized in ten‐year increments starting from age 20. Education level was categorized into three levels: low (junior high or high school), medium (technical college), and high (undergraduate or graduate school).

### 2.3 Intervention Survey

Five weeks after the baseline survey, a follow‐up survey was conducted with the same participants. In this wave, participants were randomly assigned to either an intervention group or a control group.

We developed multifaceted information about RF‐EMFs on a website. The English translation is available in the Appendix materials. First, the material introduced a national initiative conducted by Japan's Ministry of Internal Affairs and Communications (MIC): an RF‐EMF monitoring project aimed at providing the public with reliable data about RF‐EMF levels in various environments. In this project, various methods, including spot measurements at specific locations, mobile measurements using personal devices, long‐term fixed‐point measurements, and large‐scale monitoring using vehicle‐mounted equipment were employed. Following this, the main results of the monitoring project were presented, including measured RF‐EMF levels from mobile phone base stations, broadcast transmission towers, and wireless LAN equipment, along with explanations of the significance of these exposure levels.

Next, the material on four categories of information was provided: (1) the basic physics of RF‐EMFs, (2) the use of RF‐EMFs in daily life, (3) explanations of the possible adverse health effects of RF‐EMFs and guidelines, and (4) a frequently asked questions (FAQ) section with 25 common public concerns related to RF‐EMFs.

To accommodate varying levels of basic knowledge among participants, beginner‐ and intermediate‐level versions of the content were created for topics (1) and (3). The beginner‐level content targeted participants with little knowledge or interest in RF‐EMFs and presented the information in a comic‐style Q&A format to enhance accessibility. The intermediate‐level content was based on scientific literature and provided more detailed information, targeting those with greater interest or background knowledge.

In the intervention survey, participants were first shown a preview, including a picture about the MIC monitoring project and its results, along with the beginner‐level content of Section (3) as a reference. They could choose which level to view in accordance with their own preferences and literacy levels and switch between content levels freely. No time limit was imposed for reading. After viewing the content, participants completed the intervention survey, which included the same items as the baseline survey regarding attitudes toward RF‐EMFs. However, since the correct answers to 10 RF‐EMF knowledge items had been shown to participants after the baseline survey, a new set of 11 more difficult items (5 of them were related to the contents of the website) was developed for the intervention survey. During the session, participants in the intervention group were allowed to navigate the website freely, whereas participants in the control group answered the survey items directly without reading any information.

### 2.4 Statistical Analysis Plan

#### 2.4.1 Examination of Psychological Factors Related to Anxiety About RF‐EMFs

In this study, all continuous variables were z‐score standardized prior to analysis. Dietary risk sensitivity was calculated as the mean of perceived carcinogenic risk of eating red meat, eating pickled food, and drinking coffee with an acceptable Cronbach's *α* of 0.85. To investigate the psychological factors related to anxiety about RF‐EMFs, we conducted a baseline analysis based on T1 data, employing a hierarchical linear model (HLM). Anxiety about RF‐EMFs was used as the dependent variable. The independent variables included subjective exposure perceptions, exposure perception confidence, perceived necessity, awareness of RF‐EMFs, knowledge of RF‐EMFs, information exposure, information seeking, dietary risk sensitivity, gender, age, and education level. Sample ID and device type (six RF‐EMF‐based technologies) were included as random effects. These random effects account for between‐individual differences in overall anxiety levels and variation across different RF‐EMF sources, as each participant provided repeated evaluations of six devices.

#### 2.4.2 Examination of Information Provision

To evaluate the effects of information provision, we conducted an intervention analysis using data from both T1 and T2. This analysis comprised two steps, focusing respectively on the effect of information provision on the antecedent cognitive factors of anxiety about RF‐EMFs identified in the baseline model, and on anxiety itself (see Figure 2). Specifically, Step 1 examined the effect of information provision on five key antecedent cognitive factors of anxiety about RF‐EMFs. Step 2 examined its effect on anxiety at T2, while using the five cognitive factors at T2 as independent variables in the model. Since six separate regression models were used, we applied a Bonferroni‐adjusted significance threshold of *p* < 0.008 (i.e., 0.05/6).

In Step 1, for the three core RF‐EMF cognitive factors (subjective exposure perceptions, exposure perception confidence, and perceived necessity), we conducted HLM analyses using the T2 metrics as dependent variables. Independent variables included the corresponding T1 metrics, group, their interaction effect, and demographic items. Sample ID and device type were set as random intercepts. Since including random slopes for both Sample ID and device type led to convergence failure in exploratory analyses, we included random slopes only for Sample ID.

For the knowledge‐based factors (awareness and knowledge of RF‐EMFs), we conducted separate regression analyses using the same modeling structure, with T2 metrics as dependent variables. Independent variables included T1 metrics, group, their interaction effect, and demographic items.

In Step 2, we evaluated the direct effect of information provision on anxiety about RF‐EMFs after controlling for the five T2 indicators (subjective exposure perceptions, exposure perception confidence, perceived necessity, awareness of RF‐EMFs, and knowledge of RF‐EMFs). T2 anxiety served as the dependent variable. Independent variables included T1 anxiety, the five T2 indicators, group, the interaction between T1 anxiety and group, and demographic variables. Sample ID and device type were set as random intercepts, with random slopes set for Sample ID only.

## 3 Results

### 3.1 Overview

To ensure data quality, the survey company excluded responses with extremely short completion times or open‐ended answers that contained strings of random characters, resulting in an initial sample of 5007 respondents. We further excluded seven respondents who identified their gender as “other.” Additionally, 220 participants were excluded for responding “don't know” to the question “How strong is your mobile phone signal?” or selecting “don't know” for all RF‐EMF knowledge items. As a result, the final sample included 4780 responses at T1 and 1940 responses at T2, with 965 participants assigned to the intervention group and 975 to the control group.

To ensure consistency between the baseline and intervention analyses, we used only the subset of 1940 participants who completed both waves. Specifically, the T1 data from these 1940 participants were used for the baseline analysis, while both T1 and T2 data were used in the intervention analyses. The results of descriptive analysis are shown in Table S1.

### 3.2 Baseline Analysis

As planned, we conducted an HLM analysis to examine the psychological factors related to anxiety about RF‐EMFs. The variance components of the multilevel model indicated substantial between‐individual variation (sample ID intercept variance = 0.613, SD = 0.783), while the variation across device type was relatively small (type intercept variance = 0.006, SD = 0.078). The residual variance was 0.171, suggesting moderate within‐individual variability across different RF‐EMFs. The marginal *R* ^2^ was 0.205, indicating that the fixed effects alone explained about 20.5% of the variance in anxiety, while the conditional *R* ^2^ was 0.828, indicating that both fixed and random effects explained approximately 82.8% of the total variance.

The fixed effects are presented in Table 1. Among the core RF‐EMF cognitive factors, both subjective exposure perceptions and exposure perception confidence were significant positive indicators of anxiety (all *p* < 0.001), with coefficients of 0.06 and 0.08, respectively. In an additional correlation analysis, the correlation between the mean subjective exposure perception score and the mean exposure perception confidence score was small but significant (*r* = 0.143, *p* < 0.001). These suggest that in the absence of external interventions, not only the perceptions of one's level of exposure to RF‐EMFs but also the confidence in these perceptions indicate anxiety: individuals who feel more certain about their perceived exposure levels tend to experience greater anxiety. Perceived necessity also significantly predicted stronger anxiety (*p* = 0.002), indicating that individuals who view RF‐EMF‐based technologies as more necessary may feel more anxious.

**Table 1:** Results of multilevel regression analysis.

	*B*	SE	95% CI	*t* value	*p* value	${G\mathrm{VIF}}^{\frac{1}{2\mathrm{df}}}$
(Intercept)	–0.31	0.07	[–0.44, –0.18]	–4.57	< 0.001	
Subjective exposure perceptions	0.06	0.01	[0.05, 0.07]	9.43	< 0.001	1.02
Exposure perception confidence	0.08	0.01	[0.06, 0.10]	8.59	< 0.001	1.04
Perceived necessity	0.02	0.01	[0.01, 0.03]	3.03	0.002	1.00
Awareness of RF‐EMF	0.20	0.02	[0.16, 0.24]	10.53	< 0.001	1.05
Information exposure (ref = No)						1.05
Yes	0.01	0.04	[–0.06, 0.09]	0.33	0.740	
Information seeking (ref = No)						1.04
Yes	0.06	0.05	[–0.05, 0.16]	1.10	0.271	
Knowledge of RF‐EMF	–0.10	0.02	[–0.14, –0.06]	–5.20	< 0.001	1.09
Dietary risk sensitivity	0.30	0.02	[0.26, 0.34]	16.22	< 0.001	1.02
Gender (ref = Male)						1.07
Female	0.26	0.04	[0.18, 0.33]	6.65	< 0.001	
Age category (ref = 20 s)						1.01
30 s	0.09	0.06	[–0.04, 0.21]	1.38	0.166	
40 s	0.08	0.06	[–0.04, 0.19]	1.28	0.201	
50 s	0.22	0.06	[0.10, 0.33]	3.61	< 0.001	
60 s and above	0.23	0.06	[0.10, 0.35]	3.66	< 0.001	
Education level (ref = low education)						1.03
Medium education	0.03	0.06	[–0.08, 0.14]	0.61	0.545	
High education	0.06	0.04	[–0.03, 0.15]	1.34	0.181	

*Note: B* = unstandardized coefficient; *SE* = standard error; 95% CI = 95% confidence interval; *t* value = *t*‐statistic; *p* value = significance level; GVIF^1/(2df) = generalized variance inflation factor adjusted for degrees of freedom. All continuous variables were z‐score standardized prior to analysis.

For the knowledge‐based factors, awareness of RF‐EMFs showed a significant positive effect (*p* < 0.001) with a coefficient of 0.20, suggesting that individuals who are more conscious of RF‐EMFs in their environment tend to experience greater anxiety. In contrast, knowledge of RF‐EMFs had a significant negative effect (*p* < 0.001) with a coefficient of −0.10, indicating that individuals with more objective knowledge of RF‐EMFs were less likely to feel anxious. However, neither information exposure nor information seeking showed a significant effect on anxiety (*p* = 0.740 and *p* = 0.271, respectively).

Regarding individual‐specific factors, dietary risk sensitivity was significantly positively associated with anxiety, with a relatively large coefficient (*B* = 0.30). This indicates that individuals who are more sensitive to the carcinogenic potential of certain dietary behaviors also tend to experience stronger anxiety about RF‐EMFs. Among demographic variables, gender and age were significant indicators. Specifically, being female or aged 50–59 or 60 and above was associated with substantially higher levels of anxiety (all *p* < 0.001). In contrast, individuals with medium or high levels of education tended to report lower anxiety than those with lower education, but these effects were not statistically significant (*p* = 0.545 and *p* = 0.181, respectively).

Finally, to assess the robustness of these findings, we examined the difference between the 1940 participants who completed the follow‐up survey and the 2840 participants who did not on key continuous variables measured at baseline. The results indicated no significant differences between the two groups, with *p*s ranging from 0.133 to 0.729. Additionally, we conducted a sensitivity analysis using the full sample of 4780 participants from the baseline survey. As shown in Table S2, the results were highly consistent with the main analysis, suggesting that the selection of the analytical sample did not substantially bias the findings.

### 3.3 Intervention Analysis

As planned, Step 1 involved regression analyses on five antecedent cognitive factors. For the three core RF‐EMF cognitive factors, HLM analyses were conducted, and the results are detailed in Table S3. The corresponding variance components are detailed in Table S4. For the two knowledge‐based factors, multiple regression analyses were used, and the results are detailed in Table S5. The interaction effects of group and T1 metrics are illustrated in Figure 4, where the horizontal axis represents the T1 metrics and the vertical axis represents the T2 metrics.

**Figure 4:**
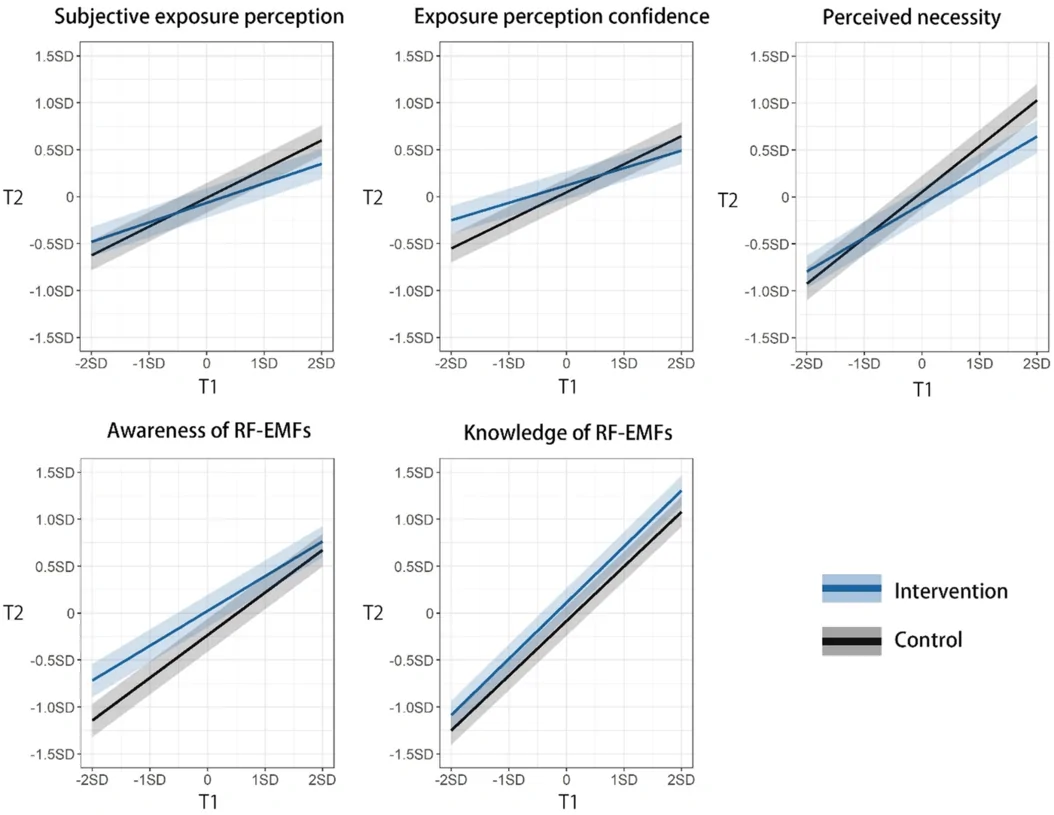
Interaction effect between information provision and antecedent factors of anxiety. The x‐axis shows the standardized score of each metric at Time 1 (T1), and the y‐axis shows the corresponding standardized score at Time 2 (T2). In the panels for subjective exposure perception, exposure perception confidence, and perceived necessity, the difference in slopes between groups indicates significant interaction effects between the T1 metric and intervention condition. This means that the effect of the intervention varied depending on the baseline level of the corresponding T1 metric.

Across all five models, a consistent pattern emerged: T1 metrics significantly predicted T2 metrics with large coefficients, indicating the persistent influence of initial cognitive states over time. For the three core RF‐EMF cognitive factors, the interaction between group and T1 metrics was significantly negative. This suggests that information provision moderated the cognitive change depending on individuals' baseline levels. Specifically, among individuals with lower T1 metrics—such as weaker subjective exposure perceptions, lower exposure perception confidence, or weaker perceived necessity—information provision led to an increase in these cognitive responses. Conversely, for individuals with higher T1 metrics, information provision slightly reduced their cognitive levels.

In contrast, for the two knowledge‐based models, the interaction terms between group and T1 metrics were not significant, with *p* values of 0.043 and 0.673. Instead, information provision showed significant positive main effects in both models (all *p* < 0.001), indicating that it led to improved awareness of RF‐EMFs in the environment and increased objective knowledge of RF‐EMFs. Importantly, these effects were independent of participants' baseline knowledge or awareness levels.

Together, these results demonstrate that information provision significantly influenced the antecedent cognitive factors of anxiety about RF‐EMFs. Among individuals who were initially unaware of RF‐EMFs in their daily lives, information provision not only increased their awareness but also correspondingly strengthened their subjective exposure perceptions. Conversely, for those who already exhibited heightened awareness and possibly excessive concern, information provision helped to moderate their cognitive responses, and while they remained conscious of RF‐EMFs in their surroundings, their subjective exposure perceptions were reduced.

In Step 2, we conducted an HLM analysis to examine the impact of information provision on anxiety about RF‐EMFs. The results are presented in Table 2, and the variance components are detailed in Table S4. The marginal *R* ^2^ was 0.294, indicating that the fixed effects alone explained about 29.4% of the variance in anxiety, while the conditional *R* ^2^ was 0.844, indicating that both fixed and random effects explained approximately 84.4% of the total variance.

**Table 2:** Indicators of T2 anxiety.

	*B*	*SE*	95% CI	*p‐*value	${G\mathrm{VIF}}^{\frac{1}{2\mathrm{df}}}$
(Intercept)	–0.15	0.06	[–0.27, –0.04]	0.008	
T1 anxiety	0.40	0.02	[0.35, 0.44]	< 0.001	1.44
T2 subjective exposure perception	0.08	0.01	[0.07, 0.10]	< 0.001	1.01
T2 exposure perception confidence	0.07	0.01	[0.05, 0.09]	< 0.001	1.03
T2 perceived necessity	0.01	0.01	[0.00, 0.02]	0.102	1.00
T2 awareness of RF‐EMFs	0.12	0.02	[0.08, 0.15]	< 0.001	1.05
T2 knowledge of RF‐EMFs	–0.10	0.02	[–0.13, –0.06]	< 0.001	1.04
Dietary risk sensitivity	0.15	0.02	[0.12, 0.18]	< 0.001	1.03
Group (ref = Control)					1.02
Intervention	0.13	0.03	[0.07, 0.20]	< 0.001	
Gender (ref = Male)					1.05
Female	0.11	0.03	[0.05, 0.18]	0.001	
Age category (ref = 20 s)					1.01
30 s	0.04	0.06	[–0.07, 0.15]	0.504	
40 s	0.02	0.05	[–0.08, 0.13]	0.698	
50 s	0.04	0.05	[–0.07, 0.14]	0.472	
60 s and above	0.08	0.05	[–0.03, 0.18]	0.161	
Education level (ref = Low education)					1.02
Medium education	0.02	0.05	[–0.08, 0.11]	0.758	
High education	0.03	0.04	[–0.05, 0.11]	0.439	
T1 anxiety × Group	–0.10	0.03	[–0.15, –0.04]	< 0.001	1.42

*Note: B* = unstandardized coefficient; *SE* = standard error; 95% CI = 95% confidence interval; *p* value = significance level; GVIF^1/(2df) = generalized variance inflation factor adjusted for degrees of freedom. All continuous variables were z‐score standardized prior to analysis.

Similar to the findings on antecedent factors, baseline anxiety showed a significant and strong positive effect on post‐intervention anxiety (*p* < 0.001), indicating high temporal stability. At T2, subjective exposure perceptions, exposure perception confidence, and awareness of RF‐EMFs all positively predicted anxiety, whereas RF‐EMF knowledge negatively predicted anxiety (all *p* < 0.001). In addition, females also tended to have greater anxiety (*p* < 0.001). These results are consistent with those of the baseline analysis. However, T2 perceived necessity did not significantly predict anxiety (*p* = 0.102), suggesting that the influence of this factor requires further investigation.

Regarding the effect of information provision, the main effect was significantly positive (*p* < 0.001). It should be noted that this coefficient reflects the direct effect of information provision on anxiety after controlling for the five T2 cognitive indicators, which were also influenced by the intervention, and therefore should not be interpreted as the total effect of information provision. This result suggests that, on average, participants in the intervention group reported higher anxiety levels at T2 than those in the control group. However, a significant negative interaction between T1 anxiety and group was also observed (*p* < 0.001). This interaction is illustrated in Figure 5, where the horizontal axis represents T1 anxiety and the vertical axis represents T2 anxiety. This indicates a differential effect: for individuals with relatively low baseline anxiety, information provision increased anxiety levels, whereas for those with extremely high anxiety, it led to a reduction.

**Figure 5:**
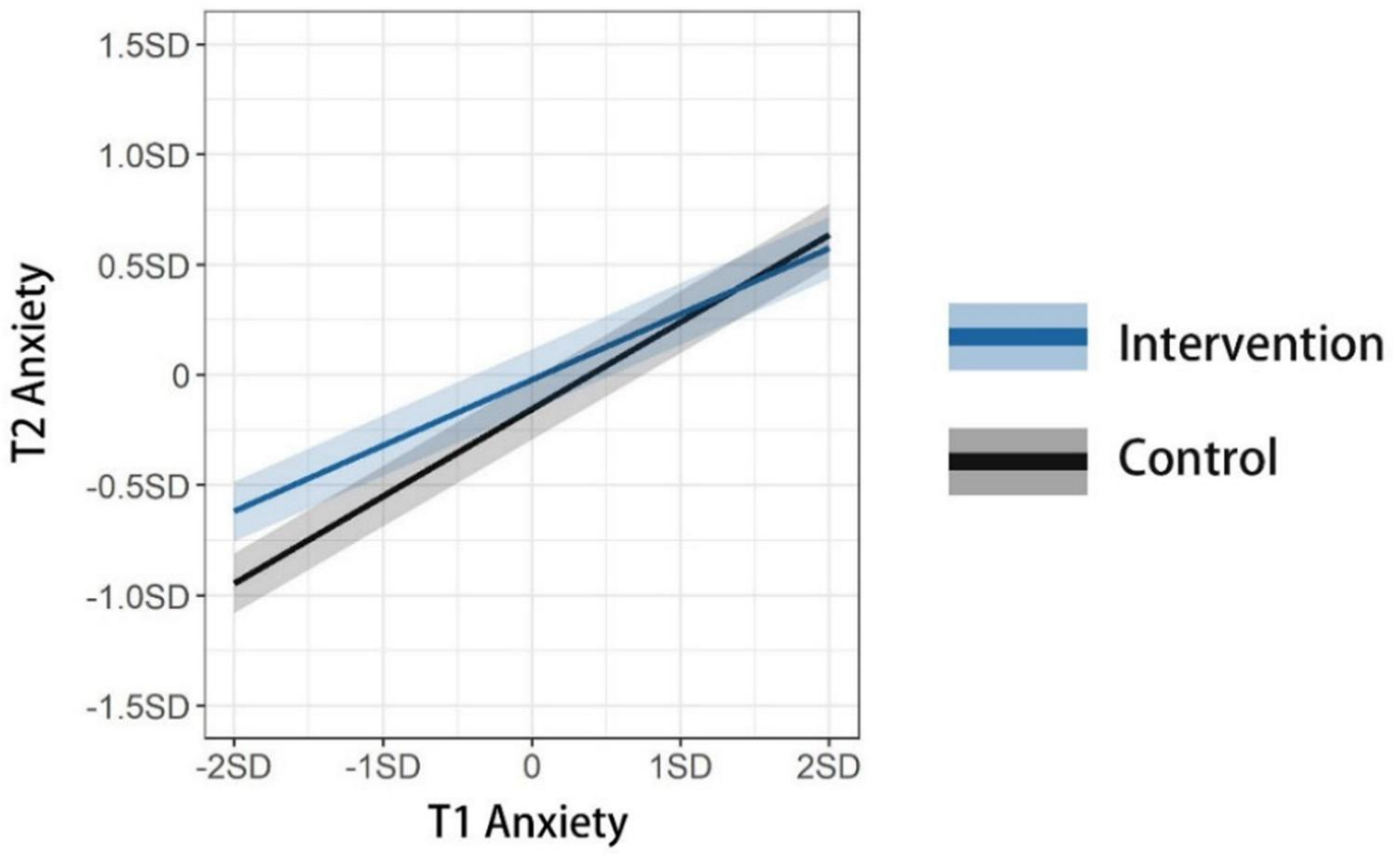
Interaction effect between information provision and T1 anxiety.

## 4 Discussion

### 4.1 Theoretical Implications

#### 4.1.1 Psychological Framework of Anxiety About RF‐EMFs

RF‐EMFs have become deeply embedded in everyday life, making it increasingly important to understand the psychological factors associated with anxiety among the general public and to explore effective risk communication strategies. In this study, we applied a multilevel modeling approach to examine anxiety about six common RF‐EMF‐based technologies in daily life. This approach accounts for the possibility that individuals may exhibit consistent cognitive tendencies across different RF‐EMF sources, thereby enabling a more accurate model fit.

The baseline HLM yielded a marginal *R* ^2^ of 0.205 and a conditional *R* ^2^ of 0.828. This pattern indicates that the fixed effects accounted for a modest proportion of the variance, whereas the considerably higher conditional *R* ^2^ largely reflects the contribution of the random effects. The remaining unexplained variance may reflect measurement error, unmodeled individual differences, and residual random variation. In particular, the variance components indicate that the intercept variance from Sample ID (0.613) was substantially larger than that from device type (0.006). This pattern suggests that variability in anxiety about RF‐EMFs was greater across individuals than across device types. One possible explanation is that, although RF‐EMFs originate from a variety of sources in daily life, the public may not differentiate clearly between specific device types when forming their perceptions. Nevertheless, recent public reactions to specific technologies, such as protests related to the rollout of 5 G (Keystone 2019), suggest that different RF‐EMF sources may not be perceived uniformly. Accordingly, it remains meaningful to consider potential variability across RF‐EMF sources, even if the associated variance component is relatively small in the present data. Future research should further examine how such variability develops, while also paying greater attention to individual differences associated with anxiety about RF‐EMFs.

Regarding psychological factors associated with anxiety about RF‐EMFs, the present study extends previous work by providing a more detailed examination of subjective exposure perception, particularly by considering individuals' confidence in these perceptions. In the baseline analysis, subjective exposure perceptions and exposure perception confidence independently predicted anxiety. This suggests that the perceptions themselves (e.g., “I am being exposed”) and the strength of conviction (e.g., “I strongly believe this” or “I am not sure about it”) are distinct dimensions that should not be conflated. Future research can further differentiate cognitive profiles—such as those who believe they are exposed but lack strong confidence, those who firmly believe they are exposed, or those who are confident they are not highly exposed—and examine how these cognitions relate to anxiety.

However, some findings call for further exploration. For example, perceived necessity also positively predicted anxiety—an unexpected finding that contrasts with conventional perspectives, which suggest that recognizing the necessity of technology typically mitigates risk perception (e.g., Slovic 1987). Nevertheless, the coefficient for perceived necessity was notably small (*B* = 0.02), indicating a limited association with anxiety and warranting cautious interpretation.

Furthermore, information exposure and information seeking were not significantly associated with anxiety. One possible reason is that, in this study, we assessed only the presence or absence of such experiences, without examining the specific content of the information encountered. Individuals may come across expert perspectives on RF‐EMFs and thereby reduce their anxiety, but they may also encounter echo chambers on social media (Sunstein 2001), which reinforce misunderstandings and amplify anxiety. As a result, these two factors may not serve as reliable indicators of anxiety. Moreover, the causal relationship between information seeking and anxiety remains uncertain. On the one hand, the information individuals access may influence their anxiety levels; on the other hand, those who already feel anxious may be more motivated to seek information to verify their concerns. In sum, future research should explore the relationships among information exposure, information seeking, and anxiety in greater detail.

In terms of individual‐specific characteristics, the findings that females and individuals aged 50 and above reported higher levels of anxiety are consistent with the results of previous research (e.g., Freudenstein et al. 2015c; Koh et al. 2020; Frey 2021). Notably, the present study also identified an interesting pattern: greater sensitivity to dietary carcinogenic risks was significantly associated with higher anxiety about RF‐EMFs. According to the IARC, RF‐EMFs were classified as Group 2B (possibly carcinogenic to humans) on the basis of limited evidence in humans. Although there is no conclusive evidence regarding the carcinogenic risks of either RF‐EMFs or those dietary behaviors, some individuals may adopt a more cautious stance toward uncertainty and perceive such risks as threatening. These findings help to clarify the psychological profile of individuals who are more likely to experience anxiety about RF‐EMFs, thereby informing the development of more targeted and effective risk communication strategies.

#### 4.1.2 Inclusive Effect of Information Provision

Building on the comprehensive psychological framework established in this study, we were able to assess the impact of information provision from a broad perspective. The findings indicate that information provision affected not only anxiety but also a range of related cognitive factors. Specifically, it significantly altered participants' subjective exposure perceptions and their confidence in those perceptions, with differential effects across subgroups: individuals with initially high levels of subjective exposure or confidence showed reductions following information provision, whereas those with initially low levels exhibited increases. In addition, information provision increased participants' awareness of RF‐EMFs in their surroundings and their overall knowledge of RF‐EMFs, regardless of baseline levels. In Step 2, these post‐intervention cognitive factors were significantly associated with anxiety. Greater awareness and stronger subjective exposure perceptions were associated with higher anxiety, whereas more objective knowledge of RF‐EMFs was associated with lower anxiety.

Notably, the participants in the intervention group reported significantly higher anxiety about RF‐EMFs. This may be because the material repeatedly referred to the biological effects and safety issues associated with RF‐EMFs. Although the materials emphasized that current exposure levels remain well within safe limits, the repeated and overly explicit use of terms such as “stimulation” and “protection guidelines” may have triggered a boomerang effect, thereby heightening emotional responses (Hartung et al. 2013). Nevertheless, information provision also reduced anxiety among those with initially extreme levels of concern. Overall, these findings highlight the effectiveness of information provision while also revealing its complex impact on anxiety and related cognitive factors, thereby offering useful guidance for the design of future risk communication strategies.

### 4.2 Practical Implications

One of the most important practical contributions of this study is the clarification of the relationship between individuals' knowledge about RF‐EMFs and their anxiety, which has direct implications for the development of effective risk communication strategies. The relationship between being informed and risk perception has long been a subject of debate. In theory, being informed of more objective information about RF‐EMFs may help to understand the safety of RF‐EMFs and thereby reduce unnecessary anxiety. This has been supported by some empirical findings (e.g., Frey 2021). However, some studies have also not revealed any significant correlation between risk perception and knowledge (e.g., Freudenstein et al. 2015a), and some even suggest that precautionary information can heighten risk perception (e.g., Wiedemann and Schütz 2005; Wiedemann et al. 2013; Boehmert et al. 2017). Indeed, the finding that information exposure and information seeking were not significant indicators of anxiety further supports the complexity of this relationship.

Regarding these results, we suggest that the general public may not simply lack information, but rather lacks access to credible and well‐designed information. In risk communication, it is important to respect the public's right to know and to transparently disclose scientific insights about RF‐EMFs (Boehmert et al. 2020). However, when conveying such scientific information, overemphasizing safety may trigger public skepticism and even provoke anxiety through a boomerang effect (Hartung et al. 2013). In contrast, our findings suggest that providing knowledge about the physical properties, operating principles, and societal necessity of technologies that use RF‐EMFs can help individuals better understand them and more accurately evaluate their biological effects, leading to the recognition that everyday levels of RF‐EMF exposure are not a cause for excessive anxiety. Future risk communication practices could build on these findings to develop more effective materials for addressing anxiety about RF‐EMFs among the general public.

Additionally, the multilevel modeling results showed that anxiety about RF‐EMFs is highly individualized. Some people in the general population are indifferent to RF‐EMFs and express little concern, while others are highly aware and anxious. This pattern is consistent with the findings of Boehmert et al. (2017), which showed that individual‐difference variables such as trait anxiety and gender may shape responses to risk communication. Together, these findings suggest that individual differences may play an important role in responses to risk communication. Accordingly, a more tailored communication approach may be beneficial. For example, those with high levels of anxiety may benefit more from reassuring messages, while those with low levels of concern may be more receptive to educational, science‐based information. Designing risk communication taking into consideration these individual psychological profiles may lead to more effective public engagement and communication outcomes.

### 4.3 Limitations

This study has the following limitations. First, we could not ensure that all participants thoroughly read the material. In an effort to balance scientific accuracy and readability, we included relatively detailed information on the RF‐EMF environment and its biological effects, resulting in lengthy content. Considering that forced reading may elicit psychological reactance (Reynolds‐Tylus 2019), we did not impose any minimum reading time; instead, participants were free to decide how long to browse the website and which content to view. As a result, the understanding and retention of the information provided through the website may vary among participants, which could have influenced the effectiveness of the information provision. The effect of reading material under more strictly controlled conditions should be explored in future research.

Second, the methodology that employed surveys online may have introduced sample bias, potentially limiting the generalizability of the findings in certain respects. Furthermore, although we suggested that anxiety about RF‐EMFs may be a global issue that transcends national and cultural boundaries, our sample consisted solely of members of the general population in Japan. Cultural differences in technology acceptance and health risk perception may influence the applicability of our findings (e.g., Masimba et al. 2019). Therefore, in future research, it should be examined whether the pattern identified in this study can be generalized across different populations.

Finally, most of the evidence obtained in the present study remains correlational, and the causality of the observed relationships is still limited. In the baseline analysis, we incorporated factors that may be associated with anxiety about RF‐EMFs; however, because this analysis was based on cross‐sectional data, it cannot provide rigorous evidence for causality. In the intervention analysis, although the effects of information provision on the post‐intervention factors can be interpreted causally, whether these post‐intervention factors mediate the effect of the intervention on post‐intervention anxiety requires further investigation. Future research should adopt more rigorous experimental designs to clarify the causal roles of these factors.

## Conflicts of Interest

The authors declare no conflicts of interest.

## Supporting information


Supporting File

## Data Availability

The data underlying this article will be shared on reasonable request to the corresponding author.
