# Supplementary material for: Does Knowing More Relate to More Anxiety? Psychological Factors Associated With Anxiety About Radiofrequency Electromagnetic Fields and the Role of Information Provision in Risk Communication
Source: Bioelectromagnetics. 2026 Oct 19;47(7):e70070. doi: 10.1002/bem.70070 (PMC13679077; doi:10.1002/bem.70070)
Supplement: Supplementary file 1 [file BEM-47-0-s001.docx]

Table S1

*Demographic items of two surveys*

| **Variables** | **Value** | **Total**  **N = 1940** | **Control Group**  **N = 965** | **Risk communication group**  **N = 975** |
| --- | --- | --- | --- | --- |
| Gender | Male | 982 | 490 | 492 |
|  | Female | 958 | 475 | 483 |
| Age | 20–29 | 314 | 158 | 156 |
|  | 30–39 | 333 | 163 | 170 |
|  | 40–49 | 438 | 216 | 222 |
|  | 50–59 | 442 | 221 | 221 |
|  | >=60 | 413 | 207 | 206 |
| Education level | Grade 1 | 506 | 243 | 263 |
|  | Grade 2 | 370 | 197 | 173 |
|  | Grade 3 | 1064 | 525 | 539 |

Table S2

*Sensitivity analysis*

|  | ***B*** | **SE** | **95 CI** | ***t* value** | ***p* value** | ${\mathbf{G}\mathbf{VIF}}^{\frac{\boldsymbol{1}}{\boldsymbol{2}\boldsymbol{df}}}$ |
| --- | --- | --- | --- | --- | --- | --- |
| (Intercept) | –0.30 | 0.05 | [–0.39, –0.20] | –6.04 | <0.001 |  |
| Subjective exposure perceptions | 0.05 | 0.00 | [0.04, 0.06] | 12.24 | <0.001 | 1.01 |
| Exposure perception confidences | 0.10 | 0.01 | [0.09, 0.11] | 16.21 | <0.001 | 1.03 |
| Perceived necessity | 0.02 | 0.00 | [0.01, 0.03] | 5.43 | <0.001 | 1.00 |
| Awareness of RF-EMF | 0.16 | 0.01 | [0.14, 0.19] | 13.35 | <0.001 | 1.06 |
| Information exposure (ref = No) |  |  |  |  |  | 1.04 |
| Yes | 0.03 | 0.03 | [–0.02, 0.08] | 1.32 | 0.188 |  |
| Information seeking (ref = No) |  |  |  |  |  | 1.04 |
| Yes | 0.07 | 0.03 | [–0.00, 0.13] | 1.96 | 0.051 |  |
| Knowledge of RF-EMF | –0.10 | 0.01 | [–0.12, –0.07] | –7.79 | <0.001 | 1.08 |
| Food risk sensibility | 0.32 | 0.01 | [0.29, 0.34] | 27.04 | <0.001 | 1.01 |
| Gender (ref = Male) |  |  |  |  |  | 1.07 |
| Female | 0.24 | 0.02 | [0.19, 0.28] | 9.54 | <0.001 |  |
| Age category (ref = 20s) |  |  |  |  |  | 1.01 |
| 30s | 0.06 | 0.04 | [–0.02, 0.14] | 1.55 | 0.120 |  |
| 40s | 0.13 | 0.04 | [0.05, 0.20] | 3.34 | <0.001 |  |
| 50s | 0.20 | 0.04 | [0.12, 0.27] | 5.17 | <0.001 |  |
| 60s and above | 0.18 | 0.04 | [0.11, 0.26] | 4.70 | <0.001 |  |
| Education level (ref = low education) |  |  |  |  |  | 1.03 |
| Medium education | 0.05 | 0.03 | [–0.02, 0.12] | 1.50 | 0.135 |  |
| High education | 0.05 | 0.03 | [–0.00, 0.11] | 1.84 | 0.066 |  |

***Note*.** *B* = unstandardized coefficient; *SE* = standard error; 95% CI = 95% confidence interval; *t* value = t-statistic; *p* value = significance level; GVIF^1/(2df) = generalized variance inflation factor adjusted for degrees of freedom. All continuous variables were z-score standardized prior to analysis.

Table S3

*Influence of risk communication and T1 metrics on T2 outcomes (multilevel data)*

|  | **Subjective exposure belief** | | | | |  | **Confidence in belief** | | | | |  | **Perceived necessity** | | | | |
| --- | --- | --- | --- | --- | --- | --- | --- | --- | --- | --- | --- | --- | --- | --- | --- | --- | --- |
|  | ***B*** | ***SE*** | **95% CI** | ***p-*value** | ${\mathbf{G}\mathbf{VIF}}^{\frac{\boldsymbol{1}}{\boldsymbol{2}\boldsymbol{df}}}$ |  | ***B*** | ***SE*** | **95% CI** | ***p-*value** | ${\mathbf{G}\mathbf{VIF}}^{\frac{\boldsymbol{1}}{\boldsymbol{2}\boldsymbol{df}}}$ |  | ***B*** | ***SE*** | **95% CI** | ***p-*value** | ${\mathbf{G}\mathbf{VIF}}^{\frac{\boldsymbol{1}}{\boldsymbol{2}\boldsymbol{df}}}$ |
| (Intercept) | –0.01 | 0.07 | [–0.15, 0.13] | 0.869 |  |  | 0.05 | 0.06 | [–0.07, 0.17] | 0.442 |  |  | 0.05 | 0.08 | [–0.11, 0.22] | 0.535 |  |
| T1 metrics | 0.31 | 0.02 | [0.28, 0.35] | <0.001 | 1.40 |  | 0.30 | 0.02 | [0.26, 0.34] | <0.001 | 1.43 |  | 0.49 | 0.02 | [0.46, 0.52] | <0.001 | 1.37 |
| Group (ref = Control) |  |  |  |  | 1.00 |  |  |  |  |  | 1.00 |  |  |  |  |  | 1.00 |
| Intervention | –0.05 | 0.03 | [–0.12, 0.01] | 0.121 |  |  | 0.07 | 0.04 | [0.00, 0.15] | 0.050 |  |  | –0.13 | 0.03 | [–0.18, –0.07] | <0.001 |  |
| Gender (ref = Male) |  |  |  |  | 1.03 |  |  |  |  |  | 1.04 |  |  |  |  |  | 1.03 |
| Female | –0.01 | 0.04 | [–0.07, 0.06] | 0.884 |  |  | –0.19 | 0.04 | [–0.27, –0.12] | <0.001 |  |  | 0.02 | 0.03 | [–0.04, 0.07] | 0.553 |  |
| Age category (ref = 20s) |  |  |  |  | 1.00 |  |  |  |  |  | 1.00 |  |  |  |  |  | 1.00 |
| 30s | 0.07 | 0.06 | [–0.05, 0.18] | 0.254 |  |  | 0.00 | 0.07 | [–0.13, 0.13] | 0.993 |  |  | –0.04 | 0.05 | [–0.13, 0.06] | 0.411 |  |
| 40s | 0.04 | 0.06 | [–0.07, 0.15] | 0.469 |  |  | 0.03 | 0.06 | [–0.09, 0.15] | 0.583 |  |  | –0.02 | 0.05 | [–0.11, 0.07] | 0.730 |  |
| 50s | 0.06 | 0.06 | [–0.05, 0.17] | 0.311 |  |  | –0.06 | 0.06 | [–0.18, 0.06] | 0.341 |  |  | –0.02 | 0.05 | [–0.11, 0.07] | 0.677 |  |
| 60s and above | 0.03 | 0.06 | [–0.08, 0.14] | 0.607 |  |  | 0.05 | 0.06 | [–0.07, 0.18] | 0.376 |  |  | –0.02 | 0.05 | [–0.11, 0.07] | 0.613 |  |
| Education level  (ref = Low education) |  |  |  |  | 1.02 |  |  |  |  |  | 1.02 |  |  |  |  |  | 1.02 |
| Medium education | –0.01 | 0.05 | [–0.11, 0.10] | 0.894 |  |  | 0.06 | 0.06 | [–0.06, 0.17] | 0.342 |  |  | –0.01 | 0.04 | [–0.09, 0.08] | 0.907 |  |
| High education | –0.02 | 0.04 | [–0.10, 0.06] | 0.693 |  |  | 0.02 | 0.04 | [–0.06, 0.11] | 0.605 |  |  | 0.05 | 0.03 | [–0.02, 0.11] | 0.174 |  |
| T1 metrics × Group | –0.10 | 0.02 | [–0.15, –0.05] | <0.001 | 1.40 |  | –0.11 | 0.03 | [–0.17, –0.06] | <0.001 | 1.42 |  | –0.13 | 0.02 | [–0.17, –0.08] | <0.001 | 1.37 |

**Note.** *B* = unstandardized coefficient; *SE* = standard error; 95% CI = 95% confidence interval; *p* value = significance level; GVIF^1/(2df) = generalized variance inflation factor adjusted for degrees of freedom. All continuous variables were z-score standardized prior to analysis.

Table S4

*Variance components for each model*

| **Random effects** |  | **Subjective exposure perceptions** | | **Exposure perception confidences** | | **Perceived necessity** | | **Anxiety** | |
| --- | --- | --- | --- | --- | --- | --- | --- | --- | --- |
| Group |  | Variance | Correlation | Variance | Correlation | Variance | Correlation | Variance | Correlation |
| Sample ID | (Intercept) | 0.48 |  | 0.59 |  | 0.29 |  | 0.41 |  |
|  | T1 metrics | 0.11 | 0.08 | 0.12 | –0.13 | 0.13 | 0.07 | 0.13 | –0.05 |
| Device type | (Intercept) | 0.01 |  | <0.01 |  | 0.03 |  | <0.01 |  |
| Residual |  | 0.31 |  | 0.15 |  | 0.30 |  | 0.15 |  |

Table S5

*Influence of risk communication and T1 metrics on T2 outcomes (non-multilevel data)*

|  | **Awareness of RF-EMFs** | | | | |  | **Knowledge of RF-EMFs** | | | | |
| --- | --- | --- | --- | --- | --- | --- | --- | --- | --- | --- | --- |
|  | ***B*** | ***SE*** | **95% CI** | ***p*-value** | ${\mathbf{G}\mathbf{VIF}}^{\frac{\boldsymbol{1}}{\boldsymbol{2}\boldsymbol{df}}}$ |  | ***B*** | ***SE*** | **95% CI** | ***p*-value** | ${\mathbf{G}\mathbf{VIF}}^{\frac{\boldsymbol{1}}{\boldsymbol{2}\boldsymbol{df}}}$ |
| (Intercept) | –0.23 | 0.07 | [–0.36, –0.10] | <0.001 |  |  | –0.08 | 0.06 | [–0.21, 0.02] | 0.158 |  |
| T1 metrics | 0.45 | 0.03 | [0.39, 0.51] | <0.001 | 1.48 |  | 0.58 | 0.03 | [0.53, 0.63] | <0.001 | 1.47 |
| Group (ref = Control) |  |  |  |  | 1.00 |  |  |  |  |  | 1.00 |
| Intervention | 0.26 | 0.04 | [0.18, 0.34] | <0.001 |  |  | 0.20 | 0.04 | [0.12, 0.27] | <0.001 |  |
| Gender (ref = Male) |  |  |  |  | 1.03 |  |  |  |  |  | 1.06 |
| Female | 0.01 | 0.04 | [–0.07, 0.10] | 0.741 |  |  | –0.03 | 0.04 | [–0.11, 0.04] | 0.382 |  |
| Age category (ref = 20s) |  |  |  |  | 1.00 |  |  |  |  |  | 1.01 |
| 30s | –0.07 | 0.07 | [–0.21, 0.06] | 0.294 |  |  | –0.01 | 0.06 | [–0.13, 0.11] | 0.872 |  |
| 40s | –0.03 | 0.07 | [–0.16, 0.10] | 0.619 |  |  | –0.02 | 0.06 | [–0.14, 0.09] | 0.700 |  |
| 50s | –0.05 | 0.07 | [–0.18, 0.08] | 0.461 |  |  | –0.05 | 0.06 | [–0.17, 0.06] | 0.380 |  |
| 60s and above | 0.09 | 0.07 | [–0.04, 0.23] | 0.159 |  |  | –0.02 | 0.06 | [–0.14, 0.10] | 0.735 |  |
| Education level  (ref = Low education) |  |  |  |  | 1.02 |  |  |  |  |  | 1.02 |
| Medium education | 0.17 | 0.06 | [0.05, 0.29] | 0.006 |  |  | –0.06 | 0.06 | [–0.16, 0.05] | 0.315 |  |
| High education | 0.15 | 0.05 | [0.05, 0.24] | 0.002 |  |  | 0.07 | 0.04 | [–0.02, 0.15] | 0.127 |  |
| T1 metrics × Group | –0.08 | 0.04 | [–0.16, 0.00] | 0.043 | 1.47 |  | 0.02 | 0.04 | [–0.06, 0.09] | 0.673 | 1.43 |

**Note.** *B* = unstandardized coefficient; *SE* = standard error; 95% CI = 95% confidence interval; *p* value = significance level; GVIF^1/(2df) = generalized variance inflation factor adjusted for degrees of freedom. All continuous variables were z-score standardized prior to analysis

**Questionnaire items English translation in the present study**

**1 Core RF-EMF cognitive factors**

**1.1 Subjective exposure perception**

*How strong do you think the radio waves emitted from the following devices are in your living environment? (Please answer based on the strength of the waves reaching the places where you usually spend time at home, not the strength emitted by each device.)
・Please select one option from "Completely absent" to "Very strong."
・If there are devices you do not use in your living environment, please write down the strength of the radio waves you imagine.
・Even if you are unsure, please write down the strength of the radio waves as you imagine it.*

|  | Completely absent | Very weak | Somewhat weak | Moderately strong | Very strong |
| --- | --- | --- | --- | --- | --- |
| Radio waves from TV/radio transmitters | □ | □ | □ | □ | □ |
| Radio wave from the wireless LAN (Wi-Fi) access point in your home | □ | □ | □ | □ | □ |
| Radio waves from mobile phones and smartphones (excluding 5G) | □ | □ | □ | □ | □ |
| Radio waves from 5G smartphones | □ | □ | □ | □ | □ |
| Microwave radiation | □ | □ | □ | □ | □ |
| Radio waves from Bluetooth wireless headphones and earphones | □ | □ | □ | □ | □ |

**1.2 Exposure perception confidence**

*Regarding the strength of the radio waves you perceive, as answered in the previous question, how confident are you in that perception? Please select the option that best matches your level of confidence.*

|  | Very confident | Somewhat confident | Not very confident | Not confident at all |
| --- | --- | --- | --- | --- |
| Radio waves from TV/radio transmitters | □ | □ | □ | □ |
| Radio waves from wireless LAN (Wi-Fi) access points within the home | □ | □ | □ | □ |
| Radio waves from mobile phones and smartphones (excluding 5G) | □ | □ | □ | □ |
| Radio waves from 5G smartphones | □ | □ | □ | □ |
| Microwave oven radio waves | □ | □ | □ | □ |
| Radio waves from Bluetooth wireless headphones and earphones | □ | □ | □ | □ |

**1.3 Perceived necessity**

*Regarding the following actions that utilize radio waves, how necessary do you think they are for yourself and your family in daily life?*

|  | Very necessary | Somewhat necessary | Not very necessary | Not necessary at all |
| --- | --- | --- | --- | --- |
| Watching TV or listening to the radio | □ | □ | □ | □ |
| Using a mobile phone or smartphone (excluding 5G) | □ | □ | □ | □ |
| Using a 5G smartphone | □ | □ | □ | □ |
| Use wireless LAN (Wi-Fi) | □ | □ | □ | □ |
| Cooking in a microwave oven | □ | □ | □ | □ |
| Using Bluetooth wireless headphones/earphones | □ | □ | □ | □ |

**1.4 Anxiety**

*As with the previous question, regarding the following actions involving radio waves, how concerned are you about the effects of radio waves on the body?*

|  | Very anxious | Somewhat anxious | Not very anxious | Not anxious at all |
| --- | --- | --- | --- | --- |
| Watching TV or listening to the radio | □ | □ | □ | □ |
| Using a mobile phone or smartphone (excluding 5G) | □ | □ | □ | □ |
| Using a 5G smartphone | □ | □ | □ | □ |
| Use wireless LAN (Wi-Fi) | □ | □ | □ | □ |
| Cooking in a microwave oven | □ | □ | □ | □ |
| Using Bluetooth wireless headphones/earphones | □ | □ | □ | □ |

**2 Knowledge-based factors**

**2.1 Awareness of RF-EMFs**

*We would like to ask about your thoughts after viewing the website. How aware are you of the radio waves around you (such as those from mobile phones, wireless LAN (Wi-Fi), TV/radio, Bluetooth products, etc.)?*

1. Not aware at all

2. Not aware

3. Somewhat aware

4. Strongly aware

**2.2 Exposure to information of RF-EMFs**

*Have you ever obtained information about radio waves (information about the nature of radio waves, their strength, safety, etc.)?*

1. I have never obtained such information in any form.

2. Have passively obtained it (e.g., happened to see it, etc.)

3. I have obtained it actively (e.g., searched for it on the Internet, etc.)

4. Both passively and actively

**2.3 Knowledge of RF-EMFs in baseline survey**

*Do you think the following statements about radio waves are correct? Please select whether you think they are correct or incorrect.*

|  | I think it is correct | Incorrect | Don't know |
| --- | --- | --- | --- |
| Radio waves travel through space | □ | □ | □ |
| The strength of radio waves generally weakens as they move away from the source | □ | □ | □ |
| When radio waves strike the body, some of their energy is absorbed within the body and converted into heat | □ | □ | □ |
| Radio waves do not occur naturally. | □ | □ | □ |
| The spread of the novel coronavirus was amplified by 5G radio waves | □ | □ | □ |
| Mobile phones and smartphones do not emit radio waves except when making calls | □ | □ | □ |
| 5G mobile phones use significantly stronger radio waves than 4G mobile phones. | □ | □ | □ |
| Microwave ovens use radio waves to heat food | □ | □ | □ |
| Television broadcast radio waves are assigned different frequency channels for each broadcasting station | □ | □ | □ |
| In mobile phones, calls are made by directly receiving the radio waves emitted by the other party's mobile phone. | □ | □ | □ |

**2.4 Knowledge of RF-EMFs in intervention survey**

*Do you think the following statements about radio waves are correct? Please select whether you think they are correct or incorrect.*

|  | I think it is correct | Incorrect | Don't know |
| --- | --- | --- | --- |
| Radio waves do not propagate in a vacuum | □ | □ | □ |
| Radio waves are also generated by natural phenomena such as lightning | □ | □ | □ |
| Radio waves have the property of propagating around obstacles | □ | □ | □ |
| All radio waves that strike the body are absorbed within it | □ | □ | □ |
| Generally, the higher the radio wave frequency, the more data can be transmitted in a given time | □ | □ | □ |
| The strength of radio waves from a mobile phone base station^※1^remains the same when measured at the same location, even after many years. | □ | □ | □ |
| Broadcast stations^※2^ The strongest signal from a broadcast station is directly beneath the transmission tower. | □ | □ | □ |
| The only factor affecting signal strength at home is what wireless devices you use inside your home. | □ | □ | □ |
| Electromagnetic waves and radio waves are different things. | □ | □ | □ |
| There are national rules established to protect the body from the effects of radio waves. | □ | □ | □ |
| Research on the cancer effects of radio waves has hardly been conducted at all. | □ | □ | □ |

※1: A mobile phone base station is a device that directly exchanges radio waves with mobile phone terminals to establish communication, relaying signals between the terminals and the telephone network.

※2: Broadcasting stations refer to transmission sites for television broadcasts (terrestrial digital broadcasting) and radio broadcasts.

**3 Individual-specific factors**

**3.1 Dietary risk sensitivity**

*We would like to ask about your thoughts after viewing the website. Using "carcinogenicity" as an example, we ask about your personal awareness of risks from various factors in daily life. For the items below, to what extent do you perceive a carcinogenic risk to yourself or your family in daily life? Please select a risk level for each.*

|  | Very high risk | High risk | Somewhat risky | Somewhat not risky | No risk | No risk at all |
| --- | --- | --- | --- | --- | --- | --- |
| Eat red meat※3 | □ | □ | □ | □ | □ | □ |
| Eating pickles | □ | □ | □ | □ | □ | □ |
| Drink coffee | □ | □ | □ | □ | □ | □ |

※3: Red meat refers to beef, pork, lamb, etc., and is distinct from "lean meat," which indicates cuts with lower fat content.

**3.2 Demographic items**

*Please tell us your gender.*

1. Male

2. Female

3. Other

*Please tell us your age.*

1. Under 20 [Excluded from survey]

2. 20s (20–29 years old)

3. 30s (30–39 years old)

4. 40s (40–49 years old)

5. 50s (50–59 years old)

6. 60s (60–69 years old)

7. 70s and older

*Please tell us your highest level of education.*

1. Junior high school

2. High School

3. Vocational school

4. Higher Vocational School

5. Technical college

6. Junior College

7. University (4-year/6-year)

8. Graduate School (Master's)

9. Graduate School (Doctoral)

10. Other (Specify: )

**Information provision in intervention survey**

The National Institute of Information and Communications Technology (NICT) plans to launch a website titled "Our Lives and Radio Wave Monitoring" to share insights about the radio waves present in our daily lives through the results of radio wave monitoring.
Please answer the following questions after viewing the screen captures from this website.
You can also view the website at the following URL:
"Our Lives and Radio Wave Monitoring": https://emc-monitoring.sakura.ne.jp/
*Note: As this website is not yet publicly available, the URL is not available now.*

**1 Screen capture of "What is Radio Monitoring?"**

*Please review this page and share your impressions based on the following questions. Areas we particularly want you to read are highlighted in yellow.*

**Japanese materials**

**English translations**

**What kind of research is this?**

- We are investigating what types of radio waves are used in our surroundings and how strong they are (see figure). This is a study commissioned by the Ministry of Internal Affairs and Communications, conducted by the National Institute of Information and Communications Technology (NICT).
- **Figure: Measurement of radio waves in various locations**

**Why is this research necessary?**

- Radio waves are invisible—they have no smell or color. Yet, devices around us make extensive use of them. We believe that if people can access concrete data about what kinds of radio waves are present in their environment and how strong they are, they will feel more confident and secure in using technologies that rely on radio waves.

**What have we learned so far?**

- So far, we have measured radio waves from mobile phone base stations (see figure), broadcasting stations, and inside homes. All of these were below the reference levels used to determine safety (radio wave protection guideline values; see note). For 5G, which began being used in 2020, the levels were more than 10,000 times lower than the guideline value.
- **Figure: Measurement results of radio waves from mobile phone base stations**
- The thick blue line represents the protection guideline value, and the solid blue line shows one ten-thousandth of that value. At all times and in all regions, the levels were below one ten-thousandth.
- (*Note*) Radio wave protection guidelines are national rules designed to prevent people from being exposed to excessive radio waves. The guideline value is the specific amount (radio wave strength) defined in these guidelines, and if exposure is below this level, there is no health impact.

**What is the goal of this research?**

- This research project aims to continue measuring data on the strength of radio waves from various sources in everyday environments until 2040 or beyond. This will allow us to respond at any time to questions about radio wave strength in daily life.

**2 Screen capture of the "Results of Radio Wave Monitoring"**

*Please review this page and share your impressions based on the following questions. Areas we particularly want you to read are highlighted in yellow.*

**Japanese materials**

**English translations**

**1. Introduction**

- Radio waves cannot be perceived by our five senses (such as sight, sound, or taste). However, it is well known that the programs sent from distant broadcasting stations reach our homes, and that we can exchange voice and data through mobile phones and wireless LAN (Wi-Fi) because of radio waves. Being able to use radio waves effectively without being aware of them is convenient, but some people feel uneasy about the radio waves around them. When people become concerned about these invisible waves, having concrete data on what kinds of radio waves are present and how strong they are can help them feel more secure in using them.
- Here, we present the results of measurements of radio wave strength in everyday environments. For basic information about radio waves, their uses, and their effects on the human body, please refer to the explanatory articles. Also, guidelines for radio wave protection, which serve as a reference for understanding radio wave strength and its impact on the human body, are useful.

**2. Radio Waves from Mobile Phone Base Stations**

- The evolution of mobile phone technology has been remarkable. Users tend to notice features such as richer functionality and larger, more beautiful displays, but there have been many other technological innovations. One of these is that, thanks to digitization and subsequent advances, communication can now occur using extremely weak radio waves. For example, in the analog era, devices were large and consumed a lot of power, limiting usage time. In contrast, digital technology has enabled smaller devices with lower power consumption and mass production.
- As radio wave usage expanded rapidly and communication volumes increased, more radio waves came into use. However, technological innovation has made it possible for individual communications to maintain high quality even with weak radio waves. This makes it difficult to simply describe trends in radio wave strength around us. Therefore, it is important to measure and investigate actual conditions.

Radio Wave Strength in Urban Areas, Suburbs, and Underground Malls

- In this research project, we measured the strength of radio waves from mobile phone base stations in urban areas and suburbs of the Tokyo metropolitan area, as well as in underground malls at major terminal stations. Although we refer to “base stations and others,” most of the radio waves measured can be considered to come from base stations. The results show that the levels are extremely low compared to the guideline values for human exposure. However, when compared to measurements taken about 10 years ago at the same locations, the levels have increased several times. We also found that radio waves are stronger in urban areas than in suburbs. Ten years ago, underground malls had almost no detectable radio waves, likely because mobile phone coverage had not yet extended there. In the current measurements, mobile phones can be used in underground malls, and the levels are about midway between those in urban and suburban areas.
- (Reference)
  Clarifying Radio Wave Strength from Mobile Phone Base Stations in Living Environments
  https://www.nict.go.jp/press/2021/12/07-1.html

Radio Wave Strength Indoors vs. Outdoors

- Mobile phone base stations are mainly installed outdoors. Indoors, some radio waves are blocked by outer walls, so levels tend to be slightly weaker than outdoors.

**3. Radio Waves from Broadcasting Stations**

- Transmission facilities at broadcasting stations vary in scale between main stations that cover wide areas and relay stations that serve areas where signals are hard to reach (see “Radio Waves Used in Daily Life”). Main stations transmit very powerful radio waves, but reception requires only weak signals. Radio waves are strongest near the source antenna and weaken with distance, but systems are designed to keep levels as uniform as possible. Here is an example of measured strength.
- The figure shows a map around the broadcasting tower in Sumida Ward and the ratio to the national guideline value. Levels are slightly higher a few hundred meters from the tower, but otherwise remain roughly the same. The power density of radio waves was similar to that from mobile phone base stations. However, because the guideline value for broadcasting frequencies is lower than that for mobile phone frequencies, radio waves from base stations were about one ten-thousandth of the guideline value, while broadcasting waves were about one several-hundredth of the guideline value.
- Measurements taken farther away, in Saitama City and Funabashi City, showed that TV broadcasting waves were about one-hundredth as strong.

**4. Radio Waves Inside Homes**

- We measured radio waves inside ordinary homes. Participating households placed small measuring devices for two days to record the strength of radio waves from mobile phone base stations, wireless LAN (Wi-Fi), and TV/FM broadcasting. The figure shows an example of indoor measurements, stacking the power densities of radio waves from mobile phone base stations, Wi-Fi, and TV/FM broadcasting.
- Some homes had most of their radio waves coming from mobile phone base stations, while others had more from Wi-Fi (mainly from routers in the home). TV/FM broadcasting waves were smaller in comparison. Since mobile phone base stations are outside the home, indoor radio wave levels are not determined solely by the devices you use. The combined strength of radio waves arriving from outside and those emitted by devices inside the home was about one thirty-thousandth of the radio wave protection guideline value (average).

**3 Screen capture of the "Electromagnetic Waves and the Body"**

*Please review this page and share your impressions based on the following questions. Areas we particularly want you to read are highlighted in yellow.*

**Japanese materials**

**English translations**

It’s been a while since we all got together like this. How has everyone been lately?

Yeah, I bought one of those trendy smart appliances. You can connect it to the internet and control it from your smartphone, even when you’re out—you can start washing or drying remotely. It’s really convenient. How about you guys?

I’ve seen those at electronics stores, but I didn’t realize they were that useful.

So that’s what’s popular now. But those things run on electromagnetic waves (see note), right? Isn’t that a bit worrying? I wonder if it’s safe for our bodies.

(Note) Electromagnetic waves include things other than radio waves, like infrared (see Q1: What’s the difference between radio waves and electromagnetic waves?), but since many people mix them up, I’m using the term this way here.

Grandma, isn’t it the same as a microwave oven? That uses electromagnetic waves too, right? We use it every day, and I’ve never worried about it.

That’s true. But with a microwave, the lid is closed, so the waves don’t seem to escape. Smart appliances, though, feel like the waves might hit your body directly. I can understand why that seems scary.

I haven’t completely ignored the health effects of radio waves either (laughs). I looked it up, and this website had a lot of details (“Our Life and Radio Wave Monitoring”). First of all, radio waves and electromagnetic waves aren’t exactly the same thing (see Q1: What’s the difference between radio waves and electromagnetic waves?). I also found that search results differ depending on whether you use “electromagnetic waves” or “radio waves.”

So what did it say?

Well, apparently, radio waves at very high levels can stimulate the body or cause heating. But there’s no solid evidence that the radio waves people worry about cause cancer.

Really? That’s reassuring.

So if we use smartphones normally, does our body heat up?

No, normally that doesn’t happen because national regulations and product design take that into account.

Exactly. There’s something called radio wave protection guidelines to prevent health effects, and devices operate at levels far below that. The standard, if I remember correctly, is 0.08 W per kilogram of body weight.

So you calculate it based on body weight?

Seems so. For me, at 60 kg, that’s 4.8 W. About the same as a small night light.

Speaking of heat, humans generate heat even when doing nothing, right? Basal metabolism for an adult male weighing 60 kg is about 1,500 kcal per day, which converts to about 72 W.

So per kilogram, that’s about 1.2 W per day. The radio wave guideline is 0.08 W per kilogram, so that’s much lower.

Looking at the website you mentioned, the actual radio waves in daily life are way below those guidelines—less than one ten-thousandth, it says. So the chance of our bodies heating up from the radio waves around us is extremely low.

But what about long-term exposure? Could that be harmful?

That’s covered on the website too. The radio wave protection guidelines are designed with long-term exposure in mind (see FAQs).

So what does that mean in the end?

There have been lots of studies on cancer risk, but even when similar surveys are done in different countries, there’s no conclusive report that radio waves cause cancer.

I’m glad we cleared that up! Thanks. Maybe we should try using smart appliances too.
